# Retraction: Radio-photothermal therapy mediated by a single compartment nanoplatform depletes tumor initiating cells and reduces lung metastasis in the orthotopic 4T1 breast tumor model

**DOI:** 10.1039/d3nr90158k

**Published:** 2023-09-01

**Authors:** Min Zhou, Jun Zhao, Mei Tian, Shaoli Song, Rui Zhang, Sanjay Gupta, Dongfeng Tan, Haifa Shen, Mauro Ferrari, Chun Li

**Affiliations:** a Department of Cancer Systems Imaging, The University of Texas MD Anderson Cancer Center, Houston, Texas 77030, USA; b The Second Affiliated Hospital of Zhejiang University, Hangzhou, Zhejiang, China; c Department of Nuclear Medicine, Renji Hospital, Shanghai Jiaotong University School of Medicine, Shanghai 200127, China; d Department of Interventional Radiology, The University of Texas MD Anderson Cancer Center, Houston, Texas 77030, USA; e Department of Pathology, The University of Texas MD Anderson Cancer Center, Houston, Texas 77030, USA; f Department of Nanomedicine, The Methodist Hospital System Research Institute, Houston, TX 77030, USA

The authors hereby wholly retract this *Nanoscale* paper due to an error in Fig. 3c and 5b.

In Fig. 3c the infrared thermal images of the mice in the control panel and the RT/PTT panel are identical.

In Fig. 5b, kidney histology of 4 four treatment groups were compared. The image used for the kidney RT/PTT panel was wrongly used and contains overlap with the kidney control panel.

We feel that these errors undermine the integrity of this study. We regret any confusion and apologize to the scientific community.

Haifa Shen and Mauro Ferrari were contacted about this retraction but did not respond to any correspondence. Signed: Min Zhou, Jun Zhao, Mei Tian, Shaoli Song, Rui Zhang, Sanjay Gupta, Dongfeng Tan, Chun Li

Date: 25/7/2023

Retraction endorsed by Heather Montgomery, Managing Editor, *Nanoscale*

